# NMDA and P2X7 Receptors Require Pannexin 1 Activation to Initiate and Maintain Nociceptive Signaling in the Spinal Cord of Neuropathic Rats

**DOI:** 10.3390/ijms23126705

**Published:** 2022-06-16

**Authors:** David Bravo, Katherine Zepeda-Morales, Carola J. Maturana, Jeffri S. Retamal, Alejandro Hernández, Teresa Pelissier, Rafael Barra, Patricio Sáez-Briones, Héctor Burgos, Luis Constandil

**Affiliations:** 1 Laboratorio de Neurobiología, Facultad de Química y Biología, Universidad de Santiago de Chile, Santiago 9170022, Chile; david.bravol@usach.cl (D.B.); katherine.zepeda@usach.cl (K.Z.-M.); jeffri.retamal@usach.cl (J.S.R.); alejandro.hernandez@usach.cl (A.H.); terepelissier@gmail.com (T.P.); 2 Center for the Development of Nanoscience and Nanotechnology (CEDENNA), Santiago 9170022, Chile; 3 Princeton Neuroscience Institute, Princeton University, Princeton, NJ 08544, USA; maturana@princeton.edu; 4 Bluestone Center, New York University, New York, NY 10010, USA; 5 Centro de Investigación Biomédica y Aplicada (CIBAP), Escuela de Medicina, Facultad de Ciencias Médicas, Universidad de Santiago de Chile, Santiago 9170022, Chile; rafael.barra@usach.cl; 6 Laboratorio de Neurofarmacología y Comportamiento, Escuela de Medicina, Facultad de Ciencias Médicas, Universidad de Santiago de Chile, Santiago 9170022, Chile; patricio.saez@usach.cl; 7 Escuela de Psicología, Facultad de Medicina y Ciencias de la Salud, Universidad Mayor, Santiago 7570008, Chile; hector.burgosg@mayor.cl

**Keywords:** pannexin 1, NMDA receptor, P2X7 receptor, neuropathic pain, wind-up

## Abstract

Pannexin 1 (Panx1) is involved in the spinal central sensitization process in rats with neuropathic pain, but its interaction with well-known, pain-related, ligand-dependent receptors, such as NMDA receptors (NMDAR) and P2X7 purinoceptors (P2X7R), remains largely unexplored. Here, we studied whether NMDAR- and P2X7R-dependent nociceptive signaling in neuropathic rats require the activation of Panx1 channels to generate spinal central sensitization, as assessed by behavioral (mechanical hyperalgesia) and electrophysiological (C-reflex wind-up potentiation) indexes. Administration of either a selective NMDAR agonist i.t. (NMDA, 2 mM) or a P2X7R agonist (BzATP, 150 μM) significantly increased both the mechanical hyperalgesia and the C-reflex wind-up potentiation, effects that were rapidly reversed (minutes) by i.t. administration of a selective pannexin 1 antagonist (10panx peptide, 300 μM), with the scores even reaching values of rats without neuropathy. Accordingly, 300 μM 10panx completely prevented the effects of NMDA and BzATP administered 1 h later, on mechanical hyperalgesia and C-reflex wind-up potentiation. Confocal immunofluorescence imaging revealed coexpression of Panx1 with NeuN protein in intrinsic dorsal horn neurons of neuropathic rats. The results indicate that both NMDAR- and P2X7R-mediated increases in mechanical hyperalgesia and C-reflex wind-up potentiation require neuronal Panx1 channel activation to initiate and maintain nociceptive signaling in neuropathic rats.

## 1. Introduction

Central sensitization is a form of pathological neuroplasticity that occurs after a lesion of the nociceptive pathway [1], characterized by molecular, cellular, synaptic and connectivity adaptations of nociceptive neural networks, which result in increased spinal neuronal signaling alongside hyperalgesia and allodynia, neuronal hyperexcitability (e.g., increased wind-up activity) and chronic neuropathic pain [2], as direct consequences of the neural lesion [3]. In the last two decades, it has been extensively reported that astrocytes and microglia also participate in spinal nociceptive synaptic transmission, acquiring relevance mainly in the modulation of nociceptive information in conditions of neuropathic pain [4,5,6].

Recently, we reported the first direct evidence of the involvement of the pannexin 1 (Panx1) channel in the spinal central sensitization process in rats with neuropathic pain [7], a concept that has subsequently been extended to other animal models of chronic pain [8,9]. Panx1 is a nonselective channel, widely expressed in the central nervous system [10,11], with high conductance in neurons [12], coexpressed with postsynaptic density 95 protein [13], and involved in glial ATP and glutamate release, among others, permeating molecules and ions [14,15,16]. Based on the prominent nociception-related behavioral and electrophysiological changes observed when Panx1 is inhibited by 10panx peptide, carbenoxolone, or probenecid, we concluded that this protein is relevant in the spinal central sensitization process in neuropathic pain, and proposed that Panx1 channels could be working as a source of inward calcium currents in dorsal horn neurons secondary to the activation of N-methyl-D-aspartate receptors (NMDARs), as described by Thompson et al. [12] for hippocampal neurons, and possibly, of P2X7 purinergic receptors (P2X7Rs) [7,17]. However, the mechanisms by which Panx1 is modulated in spinal cord cells during neuropathic pain and its interactions with pain-related ligand-dependent receptors, such as NMDARs and P2X7Rs, remain largely unclear.

Panx1 channels promote neuroinflammation in a variety of brain pathologies by mediating glutamate and ATP efflux from neurons and glia, thereby playing a crucial role in central sensitization [18]. Indeed, Panx1-mediated extracellular glutamate and ATP could activate both ionotrophic and metabotrophic signaling via NMDARs and P2XRs/P2YRs, respectively [19]. In this regard, it has been demonstrated that activation of microglial NMDARs triggers inflammation and neuronal cell death [20] and activates the NLRP3 inflammasome pathway [21], while P2X7Rs in turn give rise to noncanonical activation of the NLRP3 inflammasome, which has been linked to the pathogenesis of neuropathic pain, among other conditions [22]. Since the activation of NLRP3 inflammasome leads to IL-1 and IL-18 release, which have been found to mediate painful conditions such as neuropathic and bone cancer pain [23,24], while IL-1 can directly activate nociceptive neurons [25], it has been proposed that targeting P2X7R, upstream to inflammasomes, may more efficiently inhibit downstream cytokine cascades than therapies aimed to counteract individual cytokines [26]. Based on (i) the reported interactions between Panx1 and NMDAR or P2X7R in several pathologies of the central nervous system [17,19,27]; (ii) the evidence that Panx1 channels can release ATP [15,28] and glutamate [14,16]; and (iii) the observations that activation of NMDAR [29,30], P2X7R [31,32] and Panx1 [7,17] is mechanistically linked to hyperalgesia and synaptic potentiation (e.g., spinal wind-up) in neuropathic animals, we studied the functional interactions of NMDARs and P2X7Rs with Panx1 in the spinal cord of neuropathic animals by using behavioral and electrophysiological approaches. Since the presence of Panx1 channels in dorsal horn neurons has not yet been shown in neuropathic animals, we also assessed by confocal immunofluorescence whether Panx1 channels coexpress with the neuronal nuclei marker NeuN (or Fox-3) [33] in intrinsic dorsal horn neurons of neuropathic rats. Here, we demonstrate that activation of Panx1 channels is essential for the expression of mechanical hyperalgesia and C reflex wind-up upon activation of NMDAR- and P2X7R-dependent signaling in the spinal cord of neuropathic rats, and that Panx1 channels expressed in the intrinsic dorsal horn neurons of the rat spinal cord are probably mediating these effects.

## 2. Results

### 2.1. NMDAR-Mediated Mechanical Hyperalgesia Requires Panx1 Channel Activation to Initiate and Maintain Nociceptive Signaling in Neuropathic Rats

**Figure 1:**
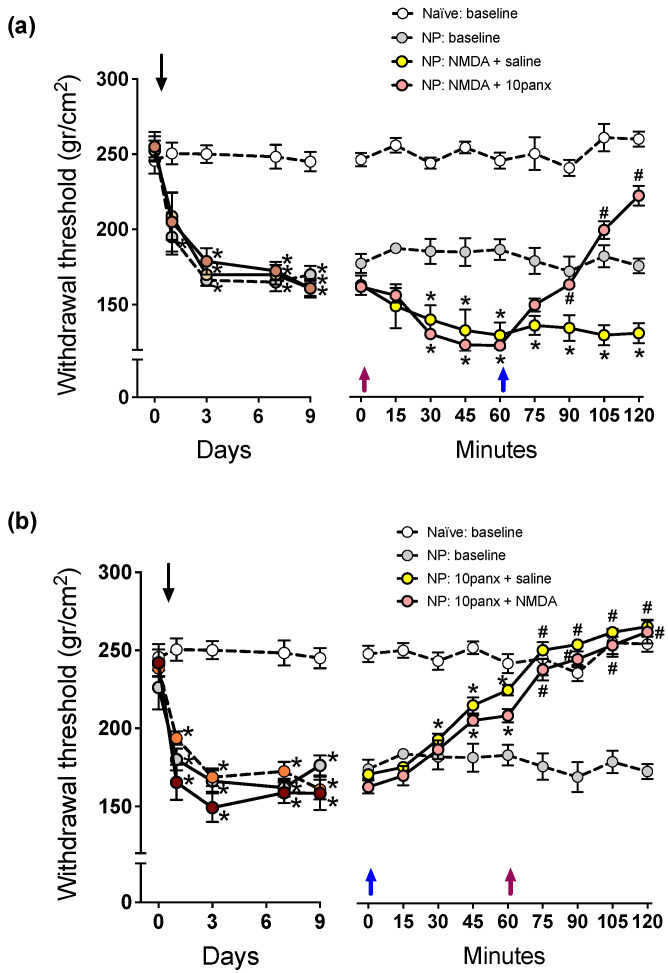
Effect of intrathecal administration of NMDA followed by 10panx, or 10panx followed by NMDA, on the mechanical nociceptive threshold (in g/cm^2^) of neuropathic (NP) rats. Naïve (baseline) and NP (baseline) rats without receiving any drug served as controls. Values are means ± SEM, *n* = 6 rats in each group. (**a**) **Left panel:** Time course of development of NP after sural nerve cutting, performed on day zero (downward arrow), during a 9-day period of follow-up. Two-way repeated measures ANOVA revealed a “time” effect (F_2.173,43.46_ = 53.40). Intragroup analysis: * *p* at least <0.05 in NP rats with respect to the threshold prior to sural nerve cutting, Bonferroni multiple comparisons test. **Right panel:** Time course of changes in mechanical nociceptive threshold on day 10, after a 10 µL injection of saline i.t. or 2 mM NMDA i.t. at time zero min (violet upward arrow), followed by saline i.t. or 300 µM 10panx i.t. at time 60 min (blue upward arrow). Two-way repeated measures ANOVA revealed a “time” effect (F_5.282,105.6_ = 15.03). Intragroup analysis: * *p* at least <0.05 with respect to time zero min, # *p* at least <0.05 with respect to time 60 min, Bonferroni multiple comparisons test). Note the hyperalgesic effect of NMDA, and the counteracting effect of 10panx. (**b**) **Left panel:** Time course of development of NP after sural nerve cutting, performed on day zero (downward arrow), during a 9-day period of follow-up. Two-way repeated measures ANOVA revealed a “time” effect (F_3.036,60.71_ = 54.54). Intragroup analysis: * *p* at least <0.05 in NP rats with respect to the threshold prior to sural nerve cutting, Bonferroni multiple comparisons test. **Right panel:** Time course of changes in the mechanical nociceptive threshold of NP rats on day 10, upon an inverse scheme of drug administration: a 10 µL injection of saline i.t. or 300 µM 10panx i.t. was administered at time zero min (blue arrow), and saline i.t. or 2 mM NMDA i.t. one hour later, at time 60 min (violet arrow). Two-way repeated measures ANOVA revealed a “time” effect (F_5.455,109.1_ = 48.38). Intragroup analysis: * *p* at least <0.05 with respect to time zero min, # *p* at least <0.05 with respect to time 60 min, Bonferroni multiple comparisons test. Note the antihyperalgesic effect of 10panx, and its preventing effect on the ability of NMDA to induce hyperalgesia.

As reported previously by Bravo et al. [7], the sural nerve section in the rat produces long-lasting mechanical hyperalgesia of the hind limb, as revealed by decreased scores in the paw pressure test (Figure 1 compares paw withdrawal scores of naïve rats versus those of neuropathic rats under i.t. saline). We evaluated first the effect of a single i.t. administration of 2 mM NMDA in the nociceptive behavior, and 1 h later, the effect of a single i.t. administration of 300 μM 10panx i.t. was tested. The results showed that i.t. NMDA (Figure 1a, violet arrow) generated a −18.3 ± 3.5% decrease in withdrawal threshold exhibiting neuropathic rats (Figure 1a, pre-NMDA injection: 163.2 ± 8.2 g/cm^2^, post-NMDA injection: 133.3 ± 5.3 g/cm^2^; * *p* < 0.05, two-way ANOVA followed by Bonferroni multiple comparisons test, *n* = 6) and, therefore, a significant increase in the mechanical hyperalgesia level which was maintained throughout the experiment, while saline did not produce any effect. Similarly, in a second group of NP rats injected with 2 mM of NMDA i.t., a significant decrease in withdrawal threshold scores was also observed, but the administration of 300 μM of 10panx 60 min later (Figure 1a, blue arrow) induced a rapid reversion of the NMDA effect, with the scores even reaching values of rats without neuropathy (Figure 1a, preinjection of NMDA: 166.2 ± 7.5 g/cm^2^, 45 min post NMDA injection: 131.5 ± 6.4 g/cm^2^, 60 min post 10panx injection: 228, 91 ± 12.1 g/cm^2^; * *p* < 0.05 with respect to time zero, #*p* < 0.05 with respect to time 60 min, two-way ANOVA followed by Bonferroni multiple comparisons test, *n* = 6), thereby indicating that the increase in mechanical hyperalgesia produced by agonist activation of NMDAR in neuropathic rats requires the opening of Panx1 channels.

In addition, the relationship between Panx1 and NMDAR was also studied inversely, where Panx1 was first inhibited and then it was studied if the activation of NMDAR can still generate hyperalgesia. The results showed that 300 μM i.t. of 10panx (Figure 1b, blue arrow) generated a significant increase in the withdrawal threshold of neuropathic rats to levels reached in rats without neuropathy (Figure 1b, preinjection of 10panx: 172.0 ± 7.2 gr/cm^2^, post-10panx injection: 210.1 ± 9.8 gr/cm^2^; * *p* < 0.05 with respect to time zero, two-way ANOVA followed by Bonferroni multiple comparisons test, *n* = 6) and, therefore, eliminated mechanical hyperalgesia. Similarly, in a second group of neuropathic rats, the i.t. injection of 10panx 300 μM significantly increased the withdrawal threshold, as in the first group. However, the subsequent application of 2 mM NMDA i.t. failed to produce its pronociceptive effects and thereby to reverse the withdrawal threshold increase produced by 10 panx (Figure 1b, preinjection of 10panx: 174.3 ± 8.4 g/cm^2^, 60 min post 10panx injection: 230.6 ± 7.1 g/cm^2^; 60 min post NMDA injection: 279.4 ± 5.2 g/cm^2^; * *p* < 0.05 with respect to time zero, # *p* < 0.05 with respect to time 60 min, two-way ANOVA followed by Bonferroni multiple comparisons test, *n* = 6). These results indicate that Panx1 channel blockade not only suppressed the mechanical hyperalgesia developed after neuropathy, but also prevented a further increase in mechanical hyperalgesia induced by pharmacological activation of NMDAR in neuropathic animals, thereby indicating that opening of Panx1 channels is essential for NMDAR-dependent nociceptive signaling can be expressed.

### 2.2. NMDAR-Evoked C-Reflex Wind-Up Requires Panx1 Channel Activation to Maintain Nociceptive Signaling in the Spinal Cord of Neuropathic Rats

**Figure 2:**
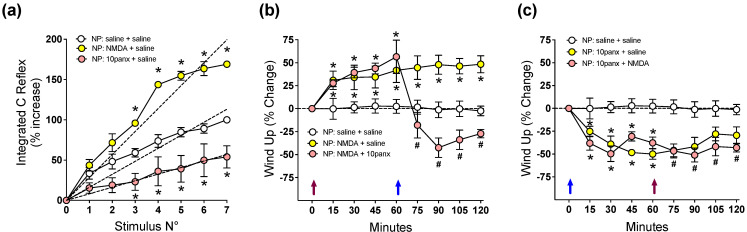
Effect of intrathecal administration of NMDA followed by 10panx, or 10panx followed by NMDA, on C-reflex wind-up activity of neuropathic (NP) rats. NP rats receiving saline served as controls. Values are means ± SEM, *n* = 6 rats in each group. (**a**) Figure depicting data processing for determining spinal cord wind-up scores in neuropathic animals. C-reflex responses were elicited by repetitive electric stimulation (1 Hz) of the second and third toes to develop wind-up potentiation, a frequency-dependent increase in the excitability of spinal cord neurons. The C-reflex responses were integrated and plotted against the stimulus number, and the curves were normalized so that the reflex gain at the seventh stimulus represented a 100% increase. The slope of the least-squares regression line represents a control wind-up score, prior to any administration of drugs or saline. Thereafter, a 10 µL i.t. injection of either saline solution (white circles), 2 mM NMDA (yellow circles) or 300 µM 10panx (pink circles) was performed, and a new series of seven repetitive electric stimulations (1 Hz) was applied 30 min after, and the wind-up scores calculated from the slopes of the respective least-squares regression lines. The procedure was repeated using 6 animals per group, and the average data is presented as Figure a. Values are means ± SEM of data averaged from 6 rats per group, and the slopes of regression curves (dashed lines) represent wind-up scores obtained 30 min after each drug treatment: 16.15 ± 0.70 (saline), 28.48 ± 0.99 * (NMDA), and 8.14 ± 0.95* (10panx), the difference between slopes being statistically significant (one-way ANOVA, F_2,15_ = 132.7; * *p* at least <0.0001 with respect to saline group, Bonferroni multiple comparisons test). (**b**) Time course of wind-up activity (expressed as % change) in neuropathic rats immediately before and 15, 30, 45, 60, 75, 90, 105, and 120 min after an i.t. injection of NMDA or saline at time 0 (violet arrow), followed by an i.t. injection of 10panx or saline at time 60 min (blue arrow). Two-way repeated measures ANOVA revealed a “time” effect (F_1.732,29.44_ = 4.504). Intragroup analysis: * *p* at least <0.05 with respect to time zero min, # *p* at least <0.05 with respect to time 60 min, Bonferroni multiple comparisons test). Note the increased wind-up activity after MDMA, and the counteracting effect of 10panx. (**c**) Time course of wind-up activity (% change) in neuropathic rats after the inverse scheme of drug administration, i.e., an i.t. injection of 10panx or saline at time 0 (blue arrow), followed by an i.t. injection of NMDA or saline at time 60 min (violet arrow). Two-way repeated measures ANOVA revealed a “time” effect (F_4.717,70.75_ = 8.484). Intragroup analysis: * *p* at least <0.05 with respect to time zero min, # *p* at least <0.05 with respect to time 60 min, Bonferroni multiple comparisons test). Note the decreased wind-up activity after 10panx, and its preventing effect on the ability of NMDA to increase wind-up.

Because central sensitization results from neuroplastic changes in the excitability of pain-transmitting neurons in the spinal cord, we tested electrophysiologically, using the C-nociceptive reflex paradigm, the effect of NMDA and 10panx on the wind-up activity of neuropathic rats, a form of plasticity in spinal dorsal horn that is observed during low-frequency electrical stimulation of C fibers. Figure 2a shows that spinal C-reflex wind-up, evoked by 1 Hz electric pulses applied to toes, significantly increased 30 min after the i.t. injection of 2 mM NMDA, as revealed by increased slope of the least-squares regression line in the saline controls, while 300 μM i.t. of 10panx induced the opposite effect. To study the interaction of NMDAR with the Panx1 channel in spinal neuronal excitability, the effect of 2 mM of NMDA i.t. on wind-up potentiation of the spinal C-reflex was first evaluated, followed by 300 μM of 10panx i.t. 60 min later. A single i.t. injection of NMDA (Figure 2b, violet arrow) generated a significant increase in wind-up activity at 15 min post injection, compared to preinjection activity, which remained increased until the end of the experiment (15 min post NMDA injection: 30.3 ± 5.7%, 120 min post NMDA injection: 48.7 ± 3.4%; * *p* < 0.05 with respect to time zero, two-way ANOVA followed by Bonferroni multiple comparisons test, *n* = 6). A second group of rats treated with 2mM NMDA (Figure 2b) also generated an increase in wind-up activity which was maintained up to 60 min post injection, but the administration of 300 μM i.t. of 10panx 60 min later induced a rapid reversion of the NMDA effect, with the scores significantly decaying even below those of the saline group for the remainder of the experiment (15 min post NMDA injection: 38.6 ± 7.3%, 60 min post NMDA injection: 52.5 ± 7.9%, 30 min post 10panx injection: −47.3 ± 9.8%; * *p* < 0.05 with respect to time zero, # *p* < 0.05 with respect to time 60 min, two-way ANOVA followed by Bonferroni multiple comparisons test, *n* = 6). These observations indicate that the NMDAR-evoked C-reflex wind-up increase requires Panx1 channel activation to maintain nociceptive signaling in the spinal cord of neuropathic rats.

To test whether NMDAR signaling requires the activation of Panx1 to initiate C-reflex wind-up potentiation, the inverse schedule of drug administration was assessed. Thus, the effect of 300 μM i.t. of 10panx followed 1 h later by i.t. administration of 2 mM NMDA was evaluated in neuropathic rats. The results showed that a single i.t. injection of 10panx (Figure 2c, blue arrow) generated a significant decrease in C-reflex wind-up activity 15 min post injection (−28.4 ± 7.7%), compared to preinjection activity, with the effect peaking at 60 min (−49.7 ± 9.2%) and maintained until the end of the experiment (120 min post injection: −47.1 ± 7.5%) (* *p* < 0.05, two-way ANOVA followed by Bonferroni multiple comparisons test, with respect to time zero). In a second group of rats, 300 μM 10panx i.t. (Figure 2c) induced a similar significant decrease in wind-up activity (−41.2 ± 7.2% at min 15 post injection, −48.1 ± 3.9% at min 60 post injection), but subsequent i.t. administration of 2mM NMDA failed to increase C-reflex wind-up and no significant difference was observed in relation to the group treated with 10panx only (−51.3 ± 9.2% at min 30 post NMDA injection). Therefore, since i.t. 10panx prevented the ability of NMDA to induce spinal wind-up, the results indicate that to initiate nociceptive signaling in the spinal cord of neuropathic rats, NMDAR depends on an open state of Panx1 channels.

### 2.3. P2X7R-Mediated Mechanical Hyperalgesia Requires Panx1 Channel Activation to Initiate and Maintain Nociceptive Signaling in Neuropathic Rats

**Figure 3:**
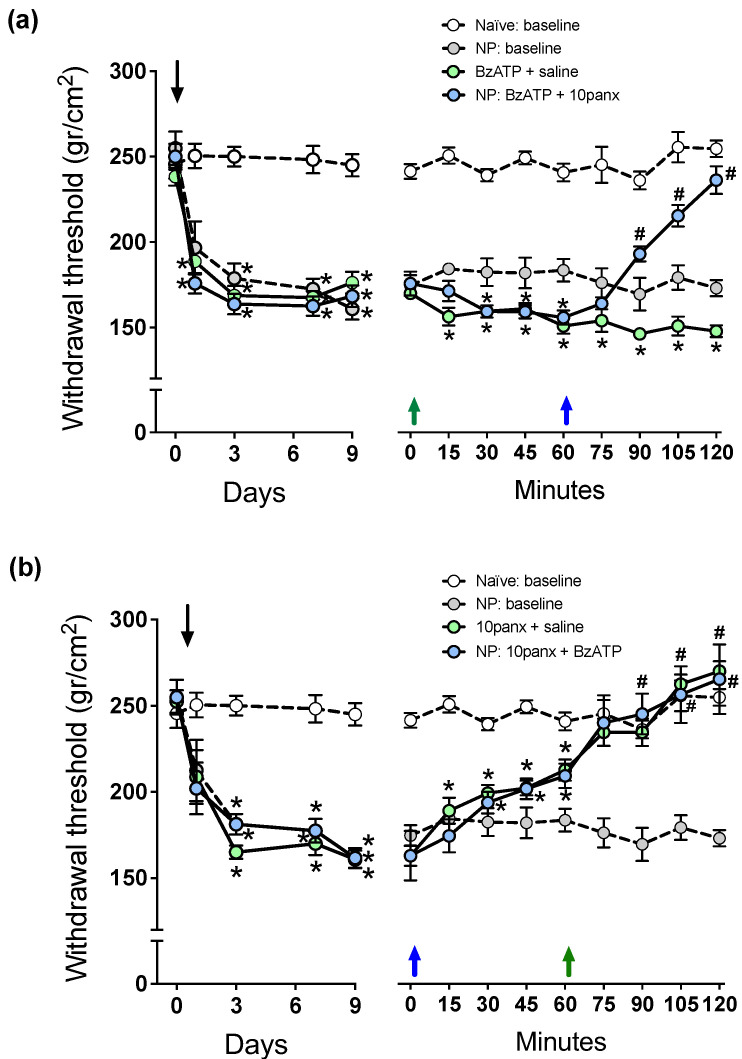
Effect of intrathecal administration of BzATP followed by 10panx, or 10panx followed by BzATP, on the mechanical nociceptive threshold (in g/cm^2^) of neuropathic (NP) rats. Naïve (baseline) and NP (baseline) rats without receiving any drug served as controls. Values are means ± SEM, *n* = 6 rats in each group. (**a**) **Left panel:** Time course of development of NP after sural nerve cutting, performed on day zero (downward arrow), during a 9-day period of follow-up. Two-way repeated measures ANOVA revealed a “time” effect (F_2.992,59.84_ = 65.12). Intragroup analysis: * *p* at least <0.05 in NP rats with respect to the threshold prior to sural nerve cutting, Bonferroni multiple comparisons test. **Right panel:** Time course of changes in mechanical nociceptive threshold after a 10 µL injection of saline i.t. or 150 µM BzATP i.t. on day 10 at time zero min (green upward arrow), followed by saline i.t. or 300 µM 10panx i.t. at time 60 min (blue upward arrow). Two-way repeated measures ANOVA revealed a “time” effect (F_5.628,112.6_ = 8.096). Intragroup analysis: * *p* at least <0.05 with respect to time zero min, # *p* at least <0.05 with respect to time 60 min, Bonferroni multiple comparisons test). Note the hyperalgesic effect of NMDA, and the counteracting effect of 10panx. (**b**) **Left panel:** Time course of development of NP after sural nerve cutting, performed on day zero (downward arrow), during a 9-day period of follow-up. Two-way repeated measures ANOVA revealed a “time” effect (F_2.112, 42.24_ = 50.46). Intragroup analysis: * *p* at least <0.05 in NP rats with respect to the threshold prior to sural nerve cutting, Bonferroni multiple comparisons test. **Right panel:** Time course of changes in the mechanical nociceptive threshold of NP rats on an inverse scheme of drug administration: a 10 µL injection of saline i.t. or 300 µM 10panx i.t. was administered at time zero min (blue arrow), and saline i.t. or 150 µM BzATP i.t. one hour later, at time 60 min (green arrow). Two-way repeated measures ANOVA revealed a “time” effect (F_5.437,108.7_ = 53.94). Intragroup analysis: * *p* at least <0.05 with respect to time zero min, # *p* at least <0.05 with respect to time 60 min, Bonferroni multiple comparisons test). Note the antihyperalgesic effect of 10panx, and its preventing effect on the ability of BzATP to induce hyperalgesia.

To test whether P2X7R interacts with Panx1 channels in spinal cord nociceptive circuitry of neuropathic rats, we first studied the effect of 150 μM i.t. administration of the P2X7R selective agonist BzATP in the paw pressure test, followed by 300 μM of 10panx i.t. 1 h later. The results showed that a single i.t. injection of BzATP (Figure 3a, green arrow) generated a decrease in withdrawal threshold of −16.2 ± 8.1%, as compared to neuropathic animals injected with saline which was maintained throughout the experiment (preinjection: 170.8 ± 9.1 g/cm^2^, 60 min post BzATP injection: 143.2 ± 8.8 g/cm^2^, 120 min post BzATP injection: 148.2 ± 11.1 g/cm^2^; * *p* < 0.05 as compared to preinjection score, two-way ANOVA followed by Bonferroni multiple comparisons test, *n* = 6), and thus a significant increase in mechanical hyperalgesia. In a second group of neuropathic rats, BzATP produced similar decreases in withdrawal threshold, but the effect was completely reverted by a 300 μM 10panx i.t. injection 60 min later (preinjection: 172.9 ± 10.1 g/cm^2^, 60 min post BzATP injection: 149.2 ± 10.5 g/cm^2^, 30 min post 10panx injection 207.3 ± 8.2 g/cm^2^; * *p* < 0.05 with respect to time zero, # *p* < 0.05 with respect to time 60 min, two-way ANOVA followed by Bonferroni multiple comparisons test, *n* = 6), with the scores even reaching values of rats without neuropathy. These observations indicate that P2X7R signaling is partially responsible for mechanical hyperalgesia but requires the opening of Panx1 channels to maintain its nociceptive signaling in neuropathic rats.

The relationship between Panx1 and P2X7R was also studied inversely, where Panx1 was first inhibited (300 μM of 10panx i.t.) and then it was studied if activation of P2X7R (150 μM BzATP i.t.) can still generate hyperalgesia in the paw pressure test. Figure 3b shows that a single i.t. injection of 10panx (blue arrow) generated an increase in withdrawal threshold of a 27.3 ± 5.2% (preinjection: 168.3 ± 9.4 g/cm^2^, 60 min post injection: 214.6 ± 8.2 g/cm^2^; * *p* < 0.05, two-way ANOVA followed by Bonferroni multiple comparisons test, *n* = 6), and thus a significant decrease in mechanical hyperalgesia, which was maintained and statistically different to the preinjection condition for the remainder of the experiment, with a peak at the end (276.8 ± 16.2 g/cm^2^). In a second group of neuropathic rats, i.t. injection of 10panx also produced a significant increase (32.3 ± 7.2%) in withdrawal threshold; however, a single i.t. injection of BzATP 60 min later (green arrow) failed to reverse the increase in the withdrawal threshold in these animals, a tendency that was maintained for the remainder of the experiment, without showing significant differences with respect the animals treated with 10panx only (# *p* > 0.05, two-way ANOVA followed by Bonferroni multiple comparisons test, *n* = 6). These results show that the Panx1 channel blockade not only decreased the basal mechanical hyperalgesia of neuropathic animals but also prevented the effect of BzATP, indicating that Panx1 is highly relevant in P2X7R signaling because when Panx1 is blocked, the pronociceptive activity of P2X7R is not observed.

### 2.4. P2X7R-Evoked C-Reflex Wind-Up Requires Panx1 Channel Activation to Maintain Nociceptive Signaling in the Spinal Cord of Neuropathic Rats

**Figure 4:**
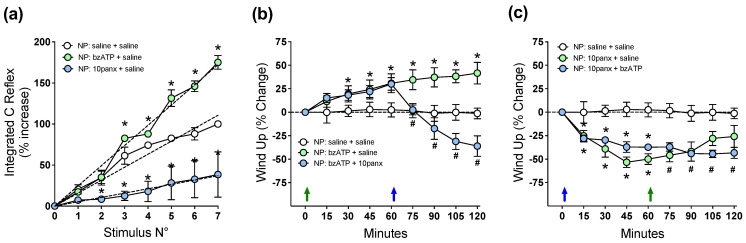
Effect of intrathecal administration of BzATP followed by 10panx, or 10panx followed by BzATP, on C-reflex wind-up activity of neuropathic (NP) rats. NP rats receiving saline served as controls. Values are means ± SEM, *n* = 6 rats in each group. (**a**) Figure depicting data processing for determining spinal cord wind-up scores in neuropathic animals, as in Figure 2a. The slope of the least-squares regression line represents a control wind-up score, prior to any administration of drugs or saline. Thereafter, a 10 µL i.t. injection of either saline solution (white circles), 150 µM BzATP (light green circles) or 300 µM 10panx (light blue circles) was performed, and a new series of seven repetitive electric stimulations (1 Hz) was applied 30 min after, and the wind-up scores calculated from the slopes of the respective least-squares regression lines. The procedure was repeated using 6 animals per group, and the average data is presented as Figure 4a. Values are means ± SEM of data averaged from 6 rats per group, and the slopes of regression curves (dashed lines) represent wind-up scores for each drug treatment: 15.80 ± 0.58 (saline), 24.62 ± 0.58 * (BzATP), and 5.28 ± 0.63 * (10panx), the difference between slopes being statistically significant (one-way ANOVA, F_2,15_ = 378.5; * *p* at least <0.0001 with respect to saline group, Bonferroni multiple comparisons test). (**b**) Time course of wind-up activity (expressed as % change) in neuropathic rats immediately before and 15, 30, 45, 60, 75, 90, 105 and 120 min after an i.t. injection of BzATP or saline at time 0 (violet arrow), followed by an i.t. injection of 10panx or saline at time 60 min (blue arrow). Two-way repeated measures ANOVA revealed a “time” effect (F_1.386,20.78_ = 3.701). Intragroup analysis: * *p* at least <0.05 with respect to time zero min, # *p* at least <0.05 with respect to time 60 min, Bonferroni multiple comparisons test). Note the increased wind-up activity after BzATP, and the counteracting effect of 10panx. (**c**) Time course of wind-up activity (% change) in neuropathic rats after the inverse scheme of drug administration, i.e., an i.t. injection of 10panx or saline at time 0 (blue arrow), followed by an i.t. injection of BzATP or saline at time 60 min (violet arrow). Two-way repeated measures ANOVA revealed a “time” effect (F_3.877,58.15_ = 8.480). Intragroup analysis: * *p* at least <0.05 with respect to time zero min, # *p* at least <0.05 with respect to time 60 min, Bonferroni multiple comparisons test. Note the decreased wind-up activity after 10panx, and its preventing effect on the ability of BzATP to increase wind-up.

Figure 4a shows that spinal C-reflex wind-up, evoked by 1 Hz electric pulses applied to toes, significantly increased 30 min after the i.t. injection of 150 μM BzATP, as revealed by the increased slope of the least-squares regression line for the saline controls, while 300 μM i.t. of 10panx induced the opposite effect. To study the interaction of P2X7R with the Panx1 channel in spinal neuronal excitability, the effect of 150 μM BzATP i.t. on wind-up potentiation of spinal C-reflex was first evaluated, followed by 300 μM of 10panx i.t. 60 min later. A single i.t. injection of BzATP (Figure 4b, green arrow) generated a significant increase in wind-up activity compared to preinjection activity (30 min post injection: 18.5 ± 3.6%, 120 min post injection: 34.6 ± 6.3%; * *p* < 0.05, two-way ANOVA followed by Bonferroni multiple comparisons test, *n* = 6). In a second group of rats, BzATP i.t. increased the wind-up activity similarly to that observed in the first one (18.2 ± 4.1% at 30 min, 30.4 ± 9.2% at 60 min; * *p* < 0.05, two-way ANOVA followed by Bonferroni multiple comparisons test, *n* = 6), but a significant decrease in wind-up potentiation was observed after injecting i.t. 10panx 60 min later (blue arrow) with respect to the preinjection condition. This decrease in wind-up activity peaked at 60 min post injection (−36.4 ± 11.7%, * *p* < 0.05, two-way ANOVA followed by Bonferroni multiple comparisons test, *n* = 6) and significant differences were maintained throughout the experiment (Figure 4b). These results indicate that P2X7R-evoked C-reflex wind-up potentiation requires Panx1 activation to maintain nociceptive signaling in the spinal cord of neuropathic rats.

To test whether P2X7R requires the activation of Panx1 to initiate a pronociceptive signaling promoting spinal wind-up potentiation, the inverse schedule of drug administration was evaluated. The results (Figure 4c) show that i.t. 10panx (blue arrow) generated a significant decrease in wind-up activity at min 15 post injection, compared to preinjection activity (−27.3 ± 9.2%; * *p* < 0.05, two-way ANOVA followed by Bonferroni multiple comparisons test, *n* = 6), with a peak at 60 min (−50 ± 5.5%), and this decrease in wind-up potentiation remained significant until the end of the experiment compared to the preinjection condition (120 min post injection: −26 ± 5.9%, * *p* < 0.05, two-way ANOVA followed by Bonferroni multiple comparisons test, *n* = 6). A similar significant decrease in wind-up activity was also observed at min 15 post injection in a second group of rats treated with 10panx i.t. (−29.5 ± 4.7%; * *p* < 0.05, two-way ANOVA followed by Bonferroni multiple comparisons test, *n* = 6, compared to preinjection condition at t = 0 min), which was maintained up to 60 min post injection (−37.3 ± 7.2%). When these 10panx-injected animals (Figure 4c) were given an i.t. injection of BzATP 60 min later (green arrow), only a nonsignificant trend to increase wind-up activity occurred (# *p* > 0.05, two-way ANOVA followed by Bonferroni multiple comparisons test, *n* = 6). These results suggest that to initiate pronociceptive spinal signaling, P2X7R depends on an open state of the Panx1 channels.

### 2.5. Pannexin Channels Are Localized in Intrinsic Dorsal Horn Neurons

**Figure 5:**
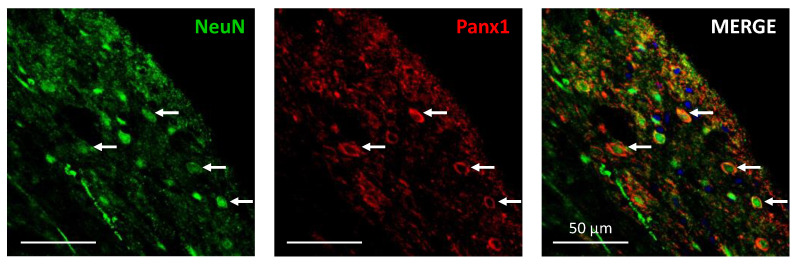
Confocal images from a 200µm-thick lumbar spinal cord transverse slice of a neuropathic rat, showing some selected neurons from Rexed laminae I and II (see white arrows) labeled with NeuN immunofluorescence (**left panel**), Panx1 immunofluorescence (**middle panel**), and double Neun/Panx1 immunofluorescence (**right panel**). Panx1 channel expression was often visualized surrounding neuronal nuclei colabeled with the NeuN protein, thus indicating the presence of Panx1 in intrinsic dorsal horn neurons. Blue labeling in the right panel corresponds to DAPI fluorescent nuclear stain. Scale bars: 50 µm in all three panels.

Confocal immunofluorescence analysis (Figure 5) showed that Panx1 channel expression was often visualized surrounding neuronal nuclei colabeled with the NeuN protein, thus indicating the presence of Panx1 in intrinsic dorsal horn neurons of neuropathic rats.

## 3. Discussion

In this study, we present evidence that, in neuropathy, NMDAR- and P2X7R-mediated pronociceptive effects are dependent on concomitant Panx1 channel activity. We firstly administrated intrathecal NMDA; then, we injected 10panx to determine if NMDAR requires the activation of the Panx1 channel to maintain its ability to induce mechanical hyperalgesia and spinal wind-up potentiation. When opened, NMDARs allow an influx of Ca^2+^, thereby activating second messengers and also depolarizing the postsynaptic membrane of the second-order nociceptive neuron in the dorsal horn, thus causing an increase in hyperalgesia and a gain in spinal wind-up activity [1,34,35]. The fact that intrathecal injection of a Panx1 blocker rapidly reverted NMDAR-mediated increments in both nociceptive behavioral activity and spinal wind-up indicates that Panx1 channels play a major role in the maintenance of NMDARs’ nociceptive signaling. Indeed, additional Ca^2+^ entry into the postsynaptic density via Panx1 likely played a relevant role, allowing Panx1 to act as an amplifier of the NMDAR activity by functioning as a Ca^2+^ permeable channel [17], as described in hippocampal neurons [19]. Mechanistically, the activation of NMDARs can downstream signalize to open Panx1 channels via the intracellular signaling of Src kinases, which phosphorylate Y308 conferring to NMDAR a modality of metabotropic signaling, whose end effector is, indeed, Panx1 [36,37]. Although metabotropic signaling of NMDAR has been described in hippocampal, cortical, retinal and striatal neurons during physiological (long-term depression and structural plasticity) and pathological (neurotoxicity and dendritic spine loss in neurodegenerative diseases) conditions [38,39], it may also be occurring in dorsal horn neurons during central sensitization in conditions of chronic neuropathic pain. The effects were reversed by 10panx, as could be seen in our behavioral and electrophysiological experiments, indicating that NMDAR cannot maintain its nociceptive signaling without Panx1 coactivation. This NMDAR–Panx1 relationship could also explain the results of the experiments involving the inverse schedule of drug administration. When Panx1, the putative effector of this downstream NMDAR-Src kinase-Panx1 pathway, is blocked, NMDAR fails to generate its characteristic nociceptive signaling, despite the fact that at the time of the experiments (10 days after neuropathy induction), NMDARs were already activated due to the neuropathic state, and also phosphorylated and with overexpressed NR2B subunits [1].

However, other alternative but feasible potential mechanisms underlying NMDAR/Panx1 functional coupling in the spinal cord of neuropathic rats could also be possible. First, Src kinase can phosphorylate tyrosine 1472 of NMDAR subunit NR2B during mechanical hyperalgesia [40], with Panx1 thus acting upstream to NMDAR signaling in this case. In this context, disrupting the interaction between Src and the NMDA receptor complex, by inhibiting the binding of kinase to the ND2 adaptor protein, reduces NMDAR-mediated uptake currents in cultured neurons and suppresses painful behavior mediated by NMDAR activation [41]. Secondly, presynaptic NMDAR activated in chronic pain states could be generating calcium inward currents, thus enhancing neurotransmitter release [42]. Since Panx1 is overexpressed in the dorsal root ganglion neurons in neuropathic pain conditions [43], perhaps it is present at the presynaptic membrane and contributes to calcium inward and neurotransmitter release, amplifying NMDAR signaling. Further investigation is required to clarify this potential mechanism.

Functional interaction of Panx1 channel with P2X7R in neuropathic animals was tested in our second experimental series. We demonstrated that the activation of P2X7R by BzATP generated increases in paw pressure threshold and spinal wind-up activity that were rapidly and fully reverted by later administration of 10panx, showing that P2X7R requires activation of Panx1 to maintain its pronociceptive signaling in conditions of chronic pain. In addition, the fact that the blockade of Panx1 completely prevented the ability of P2X7R to increase mechanical hyperalgesia and spinal wind-up potentiation in neuropathic rats demonstrates a strong and dependent functional relationship between these two transcripts in the context of chronic neuropathic pain. With regard to pain mechanisms, this functional relationship is feasible for several reasons: (i) ATP, the endogenous ligand of P2X7Rs, is released by primary nerve endings and excites superficial and deep neurons of the dorsal horn through activation of P2X purinergic receptors [44], while P2X antagonists depress wind-up in dorsal horn neurons [45]; (ii) P2X7Rs have been found to be expressed both in neurons and astrocytes of substantia gelatinosa [46], and their activation with BzATP induces robust spontaneous nociceptive behavior, which is abolished by a P2X7R antagonist [47]; (iii) P2X7R signal transduction leads to Panx1 activation through Src tyrosine kinase signaling, which is greatly attenuated by a Src tyrosine kinase inhibitor [48]; (iv) once open, Panx1 channels allow the transit of both negatively and positively charged molecules, including ions as Ca^2+^, ATP and dyes [49,50]; (v) Panx1 knockout mice (Panx1^−/−^) were protected from hypersensitivity in two sciatic nerve injury models, as Panx1^−/−^ null mice did not exhibit typical hyperalgesic and allodynic responses during neuropathy [51]. Therefore, in pain contexts, it could happen that neuronal P2X7Rs are activated by ATP released from primary nerve endings, and Src kinases would downstream open Panx1 (as in cell cultures, see Iglesias et al. [48]) that will lead to greater neuronal uptake of Ca^2+^ and release of ATP, thereby increasing both neuronal excitation and glial activation in the dorsal horn, which ultimately would lead to increased nociceptive activity.

It could be argued that the requirement of an intraneuronal P2X7R-Panx1 coupling mechanism in spinal central sensitization during neuropathic pain is controversial, because (i) it has been shown that exocyted ATP from the nociceptive nerve terminals could directly activate glial P2X7Rs, thereby opening Panx1 channels present in dorsal horn microglia, without intervention of dorsal horn neurons, at least in a chronic pain model of osteoarthritis in rats [9]; and (ii) vesicular ATP has been found to be released from dorsal horn neurons via regular exocytosis in conditions of neuropathy [52], dismissing the importance of Panx1 channels in this respect. However, the first situation is hardly compatible with the 10panx-mediated reduction in wind-up activity observed herein, because wind-up is a short-term synaptic potentiation process occurring in dorsal horn neurons, but not in glial cells. The second alternative shares the same drawback of the first, since it cannot explain the sensitivity of wind-up potentiation, which is a neuronal process, to Panx1 blockers such as 10panx, carbenoxolone or probenecid [7], thus suggesting that unlike Panx1-mediated ATP outflow, vesicular exocyted ATP from dorsal horn neurons plays a minor role in spinal cord neuronal sensitization of neuropathic animals. Furthermore, the increments of hyperalgesia and wind-up activity after activation of NMDARs or P2X7Rs and deactivation of Panx1 occurred very rapidly in the present study, within a few minutes, a timescale which seems incompatible with microglial activation and cytokine production. In fact, ATP-activated microglial release of the cytokine TNF-α became detectable only after 1 h ATP stimulation and peaked at 6 h in primary rat microglia cultures [53,54], a time course of activation that does not allow microglia to play an important role in the behavioral and electrophysiological effects described here.

Finally, functional coupling between NMDAR or P2X7R with Panx1 channels requires that these transcripts are coexpressed in the same nociceptive dorsal horn neuron. In this regard, it has been detected the existence of Panx1 channels in almost all neuronal types found in the brain [55], but the expression of Panx1 in dorsal horn neurons of the spinal cord has been rather poorly studied. Indeed, since the studies of Zappalà et al. [56] and Zhang et al. [43] failed to show the presence of the Panx1 protein in dorsal horn neurons of the spinal cord, most pain-related studies have focused on detecting and studying Panx1 channels in glial cells (for review see [57]). Here, using confocal immunofluorescence, we clearly showed coexpression of Panx1 channels (trafficking or already inserted in the neuronal somatodendritic membrane) surrounding neuronal nuclei marked with the NeuN protein in intrinsic dorsal horn neurons of neuropathic rats, thereby supporting the possibility of a Src kinase-mediated functional coupling between NMDAR and P2X7R with Panx1. It seems possible that previous negative results could arise from the fact that anti-Panx1 antibodies with epitopes against the intracellular loop or against the carboxy terminus preferentially labeled cell bodies, while others raised against an N-terminal peptide highlighted neuronal processes more than cell bodies [58].

In conclusion, we demonstrated that NMDARs and P2X7Rs required coactivation of Panx1 channels, which are expressed in intrinsic dorsal horn nociceptive neurons, to initiate and maintain mechanical hyperalgesia and C reflex wind-up activity in neuropathic rats. While not tested here, it is suggested that the coupling mechanism in the spinal cord of these animals between both NMDAR and P2X7R with Panx1 occurs in dorsal horn neurons and is mediated by an Src kinase. In contrast, participation of mediators arising from NMDA- or BzATP-activated microglia could be discarded on the basis that increments of hyperalgesia and wind-up activity occurred very rapidly within a timescale of a few minutes, incompatible with microglial activation and cytokine production. Such a concept may lead to a better understanding of the mechanisms involved in dorsal horn signaling during spinal sensitization and may help in the development of rational therapeutic approaches to the entire spectrum of neuropathies. In particular, the present results support Src kinase inhibitors as potentially useful drugs in the treatment of neuropathic pain, which needs to be tested in clinical trials.

## 4. Materials and Methods

### 4.1. Animals

Male Sprague Dawley rats weighing 225–250 g were used in the experiments. All animals were obtained from the facilities of the Faculty of Medicine of the University of Chile and were maintained with light–dark cycles of 12/12 h, starting at 8:00 AM, with food and water ad libitum. The experiments were conducted according to the NIH Laboratory Guidelines for the Care and Use of Laboratory Animals [59] and adhered to the guidelines of the IASP Committee on Research and Ethics [60]. The conditions of maintenance, the number of animals (*n* = 90 rats in total, *n* = 6 rats per group) and the experimental procedures used were approved by the Bioethics Committee of the Universidad de Santiago de Chile (protocol Nº 321).

### 4.2. Induction of Neuropathy

Neuropathy was induced by a modification of the spared injury model implemented in the rat by [61], which produces intense, prolonged, and robust changes in the mechanical and thermal sensitivity that closely match various clinical characteristics of neuropathic pain. In the present model, the right sural nerve was sectioned transversally, sparing the tibial and common peroneal nerves. This procedure allowed the generat a model of neuropathic pain [7] that preserves the reflex activity of C-fiber, since the sural nerve almost contains no motor fibers [62].

The animals were anesthetized with 400 mg/kg i.p. of a 7% (*w*/*v*) chloral hydrate solution. After shaving the right hind leg to the level of the pelvic origin of the sciatic nerve, a skin incision of approximately 10 mm long was made. The subcutaneous tissue and the biceps femoris muscle were dissected to expose the sciatic nerve. The path of the nerve was followed until its division into three branches: sural, common peroneal and tibial. The sural nerve was cut 2 mm from its separation from the sciatic, and the covering tissues were sutured in layers (NP group). During the two days following surgery, the animals were given daily 3 mg/kg s.c. of the nonsteroidal anti-inflammatory drug ketoprofen and 5 mg/kg s.c. of the antibiotic enrofloxacin. As reported earlier [7], the neuronal injury described above results in mechanical hind paw hyperalgesia that persists for at least 28 days. Another group of naïve rats, without surgery, served as control.

### 4.3. Drugs and Treatments

Panx1 channels interact with NMDARs and P2X7Rs in several neurological diseases, including neuropathic pain [17,57]. In turn, NMDAR has a crucial role in central sensitization during neuropathic pain [35,63], while P2X7R has been widely described as a crucial participant in glial activation and in the development of neuroinflammation that occurs in chronic neuropathic pain [64]. Hence, we studied the functional interaction between the Panx1 channel with NMDAR and P2X7R at the spinal cord of neuropathic rats, by using both behavioral and electrophysiological measures of nociception. For this purpose, we first evaluated the effect of a single 10 µL i.t. injection of either the selective NMDAR agonist N-methyl-D-aspartate (NMDA) 2 mM, or the selective P2X7R agonist 3′-O-(4-benzoyl) benzoyl adenosine 5′-triphosphate (BzATP) 150 μM, both on the paw pressure test and on the C-reflex wind-up paradigm. One hour later, the effect of a single i.t. administration of the selective Panx1 antagonist 10panx 300 μM was assessed. In a second series of experiments, the drug administration schedule was inverted, that is, the Panx1 antagonist was i.t. injected first, followed by the i.t. injection of the NMDAR or P2X7R agonists one hour later. The behavioral effects of NMDA, BzATP and 10panx in normal naive rats were not tested in the present study, because they were already assessed elsewhere [7,65,66]. All drugs administered (10panx, NMDA and BzATP) were from Sigma-Aldrich (St. Louis, MO, USA).

### 4.4. Behavioral Assessment of Nociceptive Behavior (Algesimetry)

Nociceptive behavior was quantified using the paw pressure test, also known as Randall–Selitto test [67]. Briefly, the right hind paw of the rat was subjected to increasing pressure by means of an algesimeter (Ugo Basile, Varese, Italy) until paw withdrawal occurred, and the threshold obtained was recorded. The maximum pressure exerted on the foot was limited to 480 g/cm^2^ (cut-off value), a pressure level that does not induce injury in the rat’s foot. This algesimetric test was performed on neuropathic rats before (day 0) and 1, 3, 7 and 9 days after surgery to verify the generation of hyperalgesia. The effects of the drugs were studied 10 days after the induction of neuropathy, by performing algesimetric measurements just before drugs (NMDA, BzATP, 10panx,) or saline injection, and then at 15, 30, 45, 60, 75, 90, 105 and 120 min after injecting the drugs or saline.

### 4.5. Electrophysiological Recording of Nociceptive C-Reflex (Electromyography)

The electromyographic activity of nociceptive reflexes transmitted by nociceptive C fibers (C-reflex) was recorded from the right femoral biceps muscle of the rats under 1.8% isoflurane in oxygen anesthesia using a latex diaphragm rodent facemask, evoked either by low-frequency (0.1 Hz, for basal C-reflex) or higher-frequency (1 Hz, for C-reflex wind-up) electric stimuli, according to [7]. All responses were analyzed offline using LabChart PowerLab software (AdInstruments, Colorado, CO, USA). Briefly, rectangular electrical pulses of supramaximal strength (double C-fiber threshold) and 2 ms duration were applied every 10 s in the receptive field of the right common peroneal and tibial nerves by means of two stainless steel needle electrodes inserted under the skin of the second and third toes of the right hind paw. The C-reflex was recorded from the ipsilateral femoral biceps muscle by using another pair of stainless-steel needle electrodes. The electromyographic responses were digitized at 100 kHz and integrated into a 150 to 450 ms time window after the stimulus. Afterward, trains of 15 stimuli each, at 1 Hz and twice the threshold intensity, were delivered to the toes to develop wind-up activity. In the C-reflex paradigm, wind-up consists of a stimulus-frequency-dependent remarkable increment of the electromyographic integrated response. Least-squares regression lines were fitted among experimental points showing only an incremental trend (before wind-up saturation at the seventh stimulus), discarding the remaining points. The slopes of the regression lines represent wind-up scores. The effect of the drugs was studied 10 days after inducing the neuropathy, by performing electrophysiological measurements just before the injection of the drugs (NMDA, BzATP, 10panx) or saline, and then at 15, 30, 45, 60, 75, 90, 105 and 120 min post injection.

### 4.6. Expression of Panx1 Protein in Dorsal Horn Neurons (Immunofluorescence)

Rats were deeply anaesthetized with chloral hydrate (1 g/kg, intraperitoneal, i.p.) and perfused transcardially with 4% paraformaldehyde in 0.1M PBS, pH 7.4. The lumbar spinal cords were removed and postfixed at 4 °C for 5 h and then transferred sequentially to 15% and 30% sucrose dissolved in PBS for 24 h. Lumbar spinal cords were transversely sliced (20 µm thick) at the levels L3-L5 in a Leica CM1520 cryostat (Leica Microsystems, Wetzlar, Germany). Floating transverse sections were incubated for 1 h in blocking solution (PBS containing 0.2% gelatin and 1% Triton X-100). Then, the slices were incubated overnight at 4 °C with either anti-Panx1 chicken polyclonal antibodies for Panx1 (1:300 dilution; Diatheva, Cartoceto, Italy) and anti-NeuN monoclonal antibody for neuronal nuclei recognition (1:300 dilution, Chemicon, Fisher Scientific, Leicestershire, UK). Samples were washed with PBS and incubated at room temperate with donkey anti-chicken Cy2 (1:300, Thermo Fisher, Waltham, MA, USA) and goat anti-mouse Alexa Fluor 647 (1:500, Thermo Fisher, Waltham, MA, USA) secondary antibodies. Samples were washed with PBS and incubated at room temperate with donkey anti-chicken Cy3 (1:300, Jackson Immuno Research Inc., West Grove, IN, USA) and goat anti-mouse Alexa Fluor 647 (1:500, Thermo Fisher, Waltham, MA, USA) secondary antibodies. Samples were mounted with DAPI-Flouromount-G (Electron Microscopy Sciences, Hatfield, PA, USA) and sections were imaged with a Nikon Eclipse Ti-E confocal microscope (Nikon Instruments, Tokyo, Japan). Stacks of consecutive images in laminae I-II of Rexed were taken with a 40× objective, and Z projections were reconstructed with ImageJ software.

### 4.7. Statistical Analysis

All data were expressed as the mean ± standard error of the mean (SEM), and statistical analyzes were performed using Prism 9.0 software (GraphPad Software Inc., San Diego, CA, USA). Time course graphs of the effect of drugs were plotted, and intragroup variations in data along time were analyzed using the two-way repeated measures ANOVA followed by the Bonferroni test for multiple comparisons. For the statistical analysis of one variable between two groups, two-tailed Student’s *t* test was used. Differences were considered significant when *p* < 0.05.

## Data Availability

Not applicable.
